# Hospitalizations among HIV controllers and persons with medically controlled HIV in the U.S. Military HIV Natural History Study

**DOI:** 10.7448/IAS.19.1.20524

**Published:** 2016-03-07

**Authors:** Trevor A Crowell, Anuradha Ganesan, Stephen A Berry, Robert G Deiss, Brian K Agan, Jason F Okulicz, for the Infectious Disease Clinical Research Program (IDCRP) HIV Working Group

**Affiliations:** 1 U.S. Military HIV Research Program, Walter Reed Army Institute of Research, Silver Spring, MD, USA; 2 Henry M. Jackson Foundation for the Advancement of Military Medicine, Bethesda, MD, USA; 3 Infectious Disease Service, Walter Reed National Military Medical Center, Bethesda, MD, USA; 4 Infectious Disease Clinical Research Program, Department of Preventive Medicine and Biostatistics, Uniformed Services University of the Health Sciences, Bethesda, MD, USA; 5 Department of Medicine, Johns Hopkins University School of Medicine, Baltimore, MD, USA; 6 Infectious Disease Clinic, Naval Medical Center San Diego, San Diego, CA, USA; 7 Infectious Disease Service, San Antonio Military Medical Center, San Antonio, TX, USA

**Keywords:** HIV, HIV non-progressors, patient admission, hospitalization, highly active antiretroviral therapy

## Abstract

**Introduction:**

HIV controllers (HICs) experience relatively low-level viraemia and CD4 preservation without antiretroviral therapy (ART), but also immune activation that may predispose to adverse clinical events such as cardiovascular disease and hospitalization. The objective of this study was to characterize the rates and reasons for hospitalization among HICs and persons with medically controlled HIV.

**Methods:**

Subjects with consistently well-controlled HIV were identified in the U.S. Military HIV Natural History Study. ART prescription and HIV-1 RNA data were used to categorize subjects as HICs or medically controlled as defined by ≥3 HIV-1 RNA measurements ≤2000 or ≤400 copies/mL, respectively, representing the majority of measurements spanning ≥12 months. Hospitalizations were tallied and assigned diagnostic categories. All-cause hospitalization rates were compared between groups using negative binomial regression.

**Results and discussion:**

Of 3106 subjects followed from 2000 to 2013, 221 were HICs, including 33 elite (1.1%) and 188 viraemic (6.0%) controllers, who contributed 882 person-years (PY) of observation time. An additional 870 subjects with medically controlled HIV contributed 4217 PY. Mean hospitalization rates were 9.4/100 PY among HICs and 8.8/100 PY among medically controlled subjects. Non-AIDS-defining infections were the most common reason for hospitalization (2.95/100 PY and 2.70/100 PY, respectively) and rates of cardiovascular hospitalization were similar in both groups (0.45/100 PY and 0.76/100 PY). There was no difference in hospitalization rate for HICs compared with subjects with medically controlled HIV (adjusted incidence rate ratio 1.15 [95% confidence interval 0.80 to 1.65]).

**Conclusions:**

All-cause and cardiovascular hospitalization rates did not differ between HICs and persons with medically controlled HIV. Non-AIDS defining infections were common in this young, healthy, predominantly male cohort of military personnel and beneficiaries.

## Introduction

HIV controllers (HICs) include both viraemic and elite controllers who are characterized by the ability to spontaneously suppress plasma HIV RNA to low or undetectable levels, respectively, in the absence of antiretroviral therapy (ART) [1–4]. These individuals naturally experience relative CD4 preservation and delayed disease progression [3,4]. However, persistent low-level viral replication in the plasma and tissues may contribute to adverse clinical events. Even elite controllers may demonstrate viraemia that is detectable by ultrasensitive assays and can exceed that which is observed among persons with well-controlled HIV on ART [5–7].

HICs have elevated markers of microbial translocation, T-cell activation, coagulation cascade activation and systemic inflammation as compared to those observed among HIV-negative persons and HIV-positive persons who are well-controlled on ART [8–11]. Separately, each of these markers has been associated with increased risk for morbidity and mortality among persons living with HIV [12–15]. These factors may contribute to the observation that elite controllers who are not prescribed ART suffer from a high burden of subclinical cardiovascular disease [16,17]. Similarly, recent evidence suggests that elite controllers may have higher rates of all-cause and cardiovascular disease-related hospitalizations than do persons with medically controlled HIV [18]. Understanding the risks of adverse clinical outcomes among HICs, including hospitalization, may inform decisions to initiate ART in this population.

The purpose of this study was to characterize the rates and reasons for hospitalization among HICs and persons with medically controlled HIV in a cohort of HIV-infected U.S. military personnel and beneficiaries.

## Methods

### Study population

The U.S. Military HIV Natural History Study (NHS) is an ongoing cohort study that has enrolled over 5800 HIV-infected military personnel and beneficiaries over the age of 18 since it began in 1986. Demographic, laboratory and clinical data are systematically collected. Subjects are seen approximately every six months. CD4 count, HIV RNA and other laboratory parameters are assessed according to the local standard of care using routine clinical assays. Hospitalization data have routinely been collected since 2000. Subjects followed from 2000 to 2013 were eligible for inclusion in this analysis. All subjects provided written informed consent prior to enrolment in the NHS, which has been approved by institutional review boards (IRBs) at participating sites. The Uniformed Services University of the Health Sciences IRB approved this analysis.

### HIV control status

HICs included both elite and viraemic controllers, as defined by at least three HIV-1 RNA measurements below the limit of detection or ≤2000 copies/mL, respectively, over a period of at least 12 months without ART, with such measurements representing the majority of measurements during the time period, as previously described [3]. The HIC period ended for elite controllers if a majority of HIV-1 measurements were detectable or if any single HIV-1 RNA measurement was ≥1000 copies/mL. The HIC period ended for viraemic controllers when the majority of HIV-1 RNA measurements were ≥2000 copies/mL. HIC periods also ended with initiation of ART. Medically controlled subjects were defined by at least three HIV-1 RNA measurements ≤400 copies/mL over a period of at least 12 months, representing the majority of measurements over that time and beginning within one year of initiating continuous ART. Medical control ended when a majority of HIV-1 RNA measurements over 12 months were >400 copies/mL or if ART was discontinued. Subjects could not contribute observation time to more than one group.

### Covariates

Race/ethnicity and sex were categorized by self-report. Age and duration of HIV infection were assessed on July 1 of each calendar year. CD4 and HIV-1 RNA were updated annually with the first available measurement.

### Outcomes

All-cause hospitalizations were tallied annually by admission date. Diagnoses were extracted at the time of data entry into the cohort database by trained study staff, using ICD-9 data and chart review to assign unique NHS diagnostic codes. The primary reason for each hospitalization was ascertained using the first-listed NHS diagnostic code. Each hospitalization was assigned to one of 17 diagnostic categories adapted from the “first-level” categories used in the Agency for Healthcare Research and Quality's Clinical Classifications Software [19]. These include system-based categories such as “cardiovascular” and “pulmonary.” These categories were modified so that specific infections and malignancies were categorized as AIDS-defining illnesses according to Centers for Disease Control and Prevention criteria [20]. Non-AIDS-defining infections and malignancies were assigned their own categories and not included in organ system categories. If NHS diagnostic codes were missing or insufficient to categorize hospitalizations, then free-text diagnosis descriptions were reviewed by two investigators who were blinded to other subject characteristics. Each investigator assigned a diagnostic category and discordant categorizations were resolved by consensus. HIV, chronic hepatitis B, chronic hepatitis C and oral candidiasis were not considered primary reasons for hospitalization since, alone, they are insufficient to justify hospitalization. If these were listed first, then the next-listed diagnosis not referring to one of these was considered the reason for hospitalization.

### Data analysis

All-cause hospitalization rates were calculated by dividing the total number of hospitalizations by the aggregate person-time during each calendar year and were reported as hospitalizations per 100 person-years (PY). Univariable and multivariable negative binomial regression models were used to estimate incidence rate ratios (IRRs) for hospitalization rates associated with pre-defined variables of interest including age, race/ethnicity, gender, first annual CD4 count and duration of HIV infection. The multivariable model also included indicators for calendar year to control for secular trends. Because hospitalizations were captured regardless of adherence to NHS study visits, years with missing CD4 data were still included in the analysis if data were available and criteria for HIV control were satisfied both before and after the missing year(s). Missing values were imputed by assuming a linear trend between known measurements up to four years apart.

All models used generalized estimating equations, clustered on subject, with exchangeable working correlation, robust variance estimators and an offset for person-time. A two-sided type I error of 5% was considered statistically significant. Analyses were performed using Stata 13.1 (StataCorp LP, College Station, TX, USA).

## Results and discussion

From 2000 to 2013, 3106 subjects contributed to the NHS database and 1091 met inclusion criteria. The HIC group consisted of 221 subjects, including 33 elite controllers (prevalence 1.1%) and 188 viraemic controllers (prevalence 6.0%), who contributed 882 PY of observation time. The medical control group included 870 subjects who contributed 4217 PY. The median age at the start of virologic control for the HIC and medical control groups was 32.2 and 33.8 years, respectively (*p*=0.025). Compared to subjects with medically controlled HIV, HICs tended to be younger at the time of HIV diagnosis (median 27.6 vs. 29.1 years, *p*<0.001), were more likely to self-identify as Black (54.3% vs. 40.1%, *p*<0.001) and female (10.9% vs. 5.3%, *p*=0.003) and had higher CD4 counts at diagnosis (median 632 vs. 422 cells/mm^3^, *p*<0.001) with lower HIV-1 RNA (median 1604 vs. 31,189 copies/mL, *p*<0.001) (Table 1).

**Table 1:** Demographic and clinical characteristics by HIV control status

Characteristics	Spontaneous HIV controllers *n*=221 (%)	Subjects with medically controlled HIV *n*=870 (%)
Age at HIV diagnosis [years]^a^		
Median (IQR)	27.6 (23.1–34.1)	29.1 (24.0–36.4)
18–29	134 (60.6)	463 (53.2)
30–44	82 (37.1)	339 (39.0)
45–59	4 (1.8)	60 (6.9)
≥ 60	1 (0.5)	8 (0.9)
Race/ethnicity		
White	75 (33.9)	347 (39.9)
Black	120 (54.3)	349 (40.1)
Hispanic	15 (6.8)	111 (12.8)
Other/unknown	11 (5.0)	63 (7.2)
Gender		
Male	197 (89.1)	824 (94.7)
Female	24 (10.9)	46 (5.3)
CD4 count at HIV diagnosis [cells/mm^3^]^b^		
Median (IQR)	632 (505–785)	422 (299–570)
> 750	65 (29.4)	78 (9.0)
501 to 750	103 (46.6)	237 (27.2)
351 to 500	40 (18.1)	254 (29.2)
201 to 350	12 (5.4)	212 (24.4)
≤ 200	1 (0.5)	89 (10.2)
HIV-1 RNA at HIV diagnosis [copies/mL]^b^		
Median (IQR)	1604 (400–5562)	31,189 (8500–91,599)
≤ 400	70 (31.7)	37 (4.3)
401 to 2000	51 (23.1)	48 (5.5)
> 2000	100 (45.2)	785 (90.2)
Duration of HIV infection at study entry [years]^c^		
< 1	84 (38.0)	208 (23.9)
1 to 3	59 (26.7)	429 (49.3)
3 to 6	30 (13.6)	159 (18.3)
6 to 10	32 (14.5)	36 (4.1)
10 to 15	13 (5.9)	31 (3.6)
> 15	3 (1.4)	7 (0.8)

IQR, interquartile range.

a Age was assessed on July 1 of the year of HIV diagnosis;

b CD4 and HIV-1 RNA measurements are the first available after HIV diagnosis;

c duration of HIV infection was assessed on July 1 of the year of entry into the study, which occurred when viral load criteria for medical or spontaneous control of HIV infection were satisfied.

A total of 455 hospitalizations were observed during 5098 PY of follow-up, including 83 among HICs and 372 among persons with medically controlled HIV. Mean hospitalization rates were 9.4/100 PY among HICs and 8.8/100 PY among medically controlled subjects. Within the HIC group, there was a trend toward fewer hospitalizations among elite controllers as compared to viraemic controllers (4.6/100 PY vs. 10.4/100 PY, respectively, *p*=0.053). Only seven hospitalizations were observed among the 33 elite controllers in this study. NHS diagnostic codes were used to assign diagnostic categories to 179 hospitalizations, and the remaining 276 hospitalizations were categorized by investigator review.

Non-AIDS-defining infection was the most common reason for hospitalization among both HICs and persons with medically controlled HIV, representing 31% of all hospitalizations. Hospitalization for non-AIDS-defining infection occurred at a rate of 2.95/100 PY among HICs and 2.70/100 PY among subjects with medically controlled HIV (Table 2). Within the 140 hospitalizations in this category, the two most common diagnoses were respiratory infections including pneumonia and influenza (37 hospitalizations) and soft tissue infections including cellulitis and abscesses (21 hospitalizations). AIDS-defining illnesses were uncommon, with only a single case of non-Hodgkin lymphoma in a medically controlled subject.

**Table 2:** Hospitalization rates by diagnostic category

	Hospitalizations per 100 person-years	
	
Diagnostic category	Spontaneous HIV controllers	Subjects with medically controlled HIV
All-cause	9.41	8.82
Non-AIDS infection	2.95	2.70
Cardiovascular	0.45	0.76
Gastrointestinal/liver	0.79	0.76
Psychiatric	0.45	0.90
Endocrine	0	0.09
Injury/poisoning	0	0.47
Renal	0.91	0.19
Pulmonary	0.34	0.07
Non-AIDS cancer	0.11	0.47
Orthopaedic	0.79	1.00
Neurologic	0.23	0.14
Symptom-based	0.79	0.45
Haematologic	0.45	0.97
AIDS-defining illness	0	0.02
Obstetric/gynaecologic	0.23	0.19
Unclassified	1.02	0.31
Dermatologic	0.23	0.07

Unadjusted and adjusted factors associated with all-cause hospitalization are presented in Table 3. Independent risk factors for hospitalization included age ≥60 (IRR 2.16 [1.01 to 4.63], as compared with 18 to 29 years) and CD4 ≤200 cells/mm^3^ (IRR 2.58 [1.46 to 4.57], as compared with >750 cells/mm^3^). There was no significant difference in hospitalization rate for HICs as compared to those with medically controlled HIV (IRR 1.15 [0.80 to 1.65]) after adjusting for age, race, sex, CD4, year and duration of HIV infection.

**Table 3:** Factors associated with all-cause hospitalization

Characteristic	Unadjusted IRR (95% CI)	Adjusted IRR (95% CI)
HIV control status		
Medical control	1.0 (Ref)	1.0 (Ref)
Spontaneous HIV control	1.07 (0.75 to 1.52)	1.15 (0.80 to 1.65)
Age [years]^a^		
18 to 29	1.0 (Ref)	1.0 (Ref)
30 to 44	0.93 (0.67 to 1.30)	0.84 (5.97 to 1.18)
45 to 59	1.48 (0.95 to 2.31)	1.14 (0.76 to 1.71)
≥ 60	**3.14 (1.58 to 6.24)**	**2.16 (1.01 to 4.63)**
Race/ethnicity		
White	1.0 (Ref)	1.0 (Ref)
Black	0.84 (0.59 to 1.22)	0.83 (0.60 to 1.14)
Hispanic	0.86 (0.55 to 1.35)	0.93 (0.60 to 1.45)
Other/unknown	0.80 (0.47 to 1.35)	0.90 (0.54 to 1.52)
Gender		
Male	1.0 (Ref)	1.0 (Ref)
Female	1.28 (0.83 to 1.98)	1.28 (0.84 to 1.95)
CD4 count [cells/mm^3^]^b^		
> 750	1.0 (Ref)	1.0 (Ref)
501 to 750	1.02 (0.73 to 1.41)	1.11 (0.79 to 1.54)
351 to 500	1.15 (0.75 to 1.76)	1.24 (0.80 to 1.95)
201 to 350	1.00 (0.64 to 1.56)	1.14 (0.72 to 1.80)
≤ 200	**2.33 (1.27 to 4.28)**	**2.58 (1.46 to 4.57)**
Duration of HIV infection [years]^c^		
< 1	1.0 (Ref)	1.0 (Ref)
1 to 3	1.34 (0.70 to 2.54)	0.77 (0.41 to 1.44)
3 to 6	1.23 (0.64 to 2.36)	0.65 (0.34 to 1.22)
6 to 10	1.26 (0.66 to 2.40)	0.63 (0.33 to 1.19)
10 to 15	1.62 (0.82 to 3.18)	0.72 (0.36 to 1.42)
> 15	**3.50 (1.55 to 7.92)**	1.43 (0.62 to 3.32)

IRR, incidence rate ratio; CI, confidence interval. Results in bold are statistically significant (*p*≤0.05). The multivariable model also included an indicator variable for calendar year to control for secular trends. Results in bold are considered statistically significant (*p*≤0.05).

a Age was assessed on July 1 of each calendar year;

b CD4 measurements used in this analysis were the first available measurements for each calendar year

c duration of HIV infection was assessed on July 1 of each calendar year.

This analysis reveals no difference in hospitalization rates between subjects with spontaneous virologic control of HIV and those with HIV that is well controlled using ART. Non-AIDS defining infections were the most common reason for hospitalization in both groups. Overall, hospitalization was relatively uncommon in this HIV-positive population with well-controlled disease and access to the U.S. military healthcare system.

Our primary finding is consistent with a prior secondary analysis of data from the NHS that showed no difference in the proportion of HICs hospitalized, as compared to other subjects in the cohort [3]. In our updated analysis, no difference was observed despite a larger sample size, more defined comparator group with medically controlled disease and statistical adjustments for confounding variables and differences in observation time. This is in contrast to a recent investigation of hospitalizations among elite controllers that found increased rates of all-cause, cardiovascular and psychiatric hospitalizations as compared to persons with medically controlled HIV [18]. These contrasting findings may be partially due to distinct virologic and immunologic profiles between elite controllers and viraemic controllers [21–23]. The latter constituted the majority of HICs in this study, resulting in an HIC population that may be phenotypically different from the elite controller population previously studied. This study was not adequately powered to detect a difference in hospitalization rates between elite controllers and subjects with medically controlled HIV. Furthermore, the military cohort evaluated here may have been too young to detect a difference in hospitalization rates driven by age-related diagnoses such as cardiovascular disease.

Non-AIDS-defining infections were the most common reason for hospitalization in both groups examined in this study. Targeted interventions should be pursued, such as vaccinations against influenza and pneumococcal pneumonia, in order to potentially reduce morbidity and hospitalizations [24,25]. There was no suggestion of a trend toward more cardiovascular hospitalizations among HICs despite prior evidence of a high burden of cardiovascular disease in this population [16–18].

The overall hospitalization rates among HICs and persons with medically controlled HIV were low compared with other reports of hospitalizations among HIV-positive individuals. Recent reports have estimated all-cause hospitalization rates among persons living with HIV in the United States as 20.6 to 28.9 hospitalizations/100 PY [26–28]. These cohorts reported mean and median ages of about 45 years. By contrast, from 1999 to 2007, the overall hospitalization rate in the younger population of the NHS was only 13.7/100 PY [29]. Hospitalization rates observed in this study are lower than those observed in the general NHS population and are higher than the all-cause hospitalization rate among active duty U.S. military personnel, which was 5.5/100 PY in 2013 [30]. Low hospitalization rates in the military and NHS may make any difference in rates between HICs and subjects with medically controlled HIV more difficult to detect.

Differences between our findings and those reported in other cohorts may partially be explained by unique characteristics of the NHS, which predominantly includes young, healthy men with universal access to healthcare. The HICs in this analysis were roughly two decades younger than those in previous investigations, where mean and median ages ranged from 45 to 52 years [16–18]. This difference is especially important when considering the effects of age-related comorbidities, such as cardiovascular disease. Also, comorbidities are generally uncommon among subjects in the NHS, including those known to be associated with high hospitalization rates such as viral hepatitis [31–33].

Our study has several strengths. Universal access to healthcare reduces the possibility of selection bias based on access to care that is observed in other cohorts. This also minimizes the risk for incomplete capture of hospitalization data. Since military personnel are regularly screened for HIV, seroconversion data are available for most participants in the NHS, and we were able to control for duration of HIV infection in statistical models.

Our study is limited by the small sample size of HICs. Because of this, we were unable to perform statistical tests for comparisons of cause-specific hospitalizations and could not meaningfully compare hospitalizations between elite and viraemic controllers. Data on tobacco use were not available for inclusion in multivariable models and this factor could have influenced the results of this analysis. Certain variables known to be associated with hospitalization could not be included in multivariable models because they are too uncommon in the cohort. For example, the rate of injection drug use in the cohort is under 1%, partially due to the periodic random drug testing that occurs among military personnel [34]. The prevalence of chronic hepatitis B and/or C is only about 6% each [33]. The unique demographic characteristics of the NHS may limit generalizability of our findings to other populations of persons living with HIV.

## Conclusions

Hospitalization rates did not differ between subjects with spontaneous virologic control of HIV and those with medically controlled HIV on ART. Non-AIDS defining infections were common and no difference in hospitalization rates related to cardiovascular disease was detected between HICs and persons with medically controlled HIV. The overall hospitalization rate in this young, healthy cohort of military personnel and beneficiaries was lower than that observed in other cohorts of persons living with HIV. Longer term follow-up of these subjects is needed, particularly to assess outcomes that are associated with aging, such as the development of cardiovascular disease.
